# The Disproportionate High Risk of HIV Infection Among the Urban Poor in Sub-Saharan Africa

**DOI:** 10.1007/s10461-012-0217-y

**Published:** 2012-06-04

**Authors:** Monica A. Magadi

**Affiliations:** Department of Sociology, School of Social Sciences, City University, Northampton Square, London, EC1V 0HB UK

**Keywords:** HIV prevalence, Urban poverty, Sub-Saharan Africa, Demographic and health surveys, Multilevel logistic regression, Gender disparity

## Abstract

The link between HIV infection and poverty in sub-Saharan Africa (SSA) is rather complex and findings from previous studies remain inconsistent. While some argue that poverty increases vulnerability, existing empirical evidence largely support the view that wealthier men and women have higher prevalence of HIV. In this paper, we examine the association between HIV infection and urban poverty in SSA, paying particular attention to differences in risk factors of HIV infection between the urban poor and non-poor. The study is based on secondary analysis of data from the Demographic and Health Surveys from 20 countries in SSA, conducted during 2003-2008. We apply multilevel logistic regression models, allowing the urban poverty risk factor to vary across countries to establish the extent to which the observed patterns are generalizable across countries in the SSA region. The results reveal that the urban poor in SSA have significantly *higher* odds of HIV infection than their urban non-poor counterparts, despite poverty being associated with a significantly *lower* risk among rural residents. Furthermore, the gender disparity in HIV infection (i.e. the disproportionate higher risk among women) is amplified among the urban poor. The paper confirms that the public health consequence of urban poverty that has been well documented in previous studies with respect to maternal and child health outcomes does apply to the risk of HIV infection. The positive association between household wealth and HIV prevalence observed in previous studies largely reflects the situation in the rural areas where the majority of the SSA populations reside.

## Introduction

The world’s poorest region, sub-Saharan Africa (SSA), remains disproportionately affected by HIV/AIDS, accounting for about two-thirds of HIV infections worldwide and almost three-quarters of global AIDS-related deaths in 2010 [1]. The relationship between HIV/AIDS and poverty is rather complex. Although the link between HIV/AIDS and poverty in SSA has received considerable research attention, findings from existing research remain inconsistent. While some argue that poverty increases individual vulnerability to HIV infection [2–4], empirical evidence largely suggest that men and women living in wealthier households have higher HIV prevalence than those living in poorer ones [5–8]. Studies based on other indicators of socio-economic status (SES) also support the view that the risk is likely to be higher among higher SES groups. For instance, evidence from five countries in East and West Africa suggest a positive education gradient in HIV infection [9]. It has been noted that credible evidence exists for both arguments: while wealth shows an increased risk for both sexes, poverty places women at a special disadvantage [10].

One possible explanation for the positive association between HIV and poverty is grounded in the theory of economics of sexual behavior—the adverse future life chances of people living in poverty are likely to increase their readiness to take risks today [11, 12]. In particular, women living in deprived urban settings have been observed to engage in riskier sexual behavior than their counterparts in less deprived areas [13]. For women living in poverty, increased vulnerability has been attributed to possible interaction between poverty and non-biological factors such as gender-based violence and transactional sex [10, 14, 15].

A number of explanations have been proposed for the apparent higher prevalence of HIV among those of higher SES [5, 11]. It has been argued that being wealthier may lead to reckless lifestyle and risky sexual relationships as wealthier people (particularly men) tend to attract multiple partners [16–18]. Shelton and others noted that wealth and social interaction are inextricably linked, and wealth might increase the number of opportunities for concurrent heterosexual partnerships to develop [5].

Another possible explanation for the positive association between wealth and HIV infection relates to higher household wealth among urban residents, and higher HIV prevalence in urban areas [5]. While this explanation may hold for associations based on bivariate relationships, the positive association has been observed to persist even after controlling for urban/rural residence in multivariate analyses [19], implying that there exists other important explanations. Also, it is possible that the longer survival of wealthier individuals infected with HIV (HIV prevalence being partly a function of survival [5]) may indeed induce a positive association between wealth and HIV.

The positive association between household wealth and HIV prevalence observed in previous studies in SSA is inconsistent with findings for other public health outcomes which have been shown to be relatively poor among lower SES sub-groups of the population, especially in urban settings. The public health consequences of urban poverty under conditions of rapid urban growth have been well documented [20–23], but there has been scant comparative research on reproductive health inequalities in urban areas, especially with respect to HIV/AIDS in SSA. Although previous studies highlight the importance of location as a risk factor for HIV incidence in urban settings [24–26], little is known about the link between urban poverty and HIV infection in SSA.

In this paper, we examine the association between urban poverty and the risk of HIV infection. The analysis places particular emphasis on the variation in the urban poverty risk factor in HIV infection across countries in SSA to establish the extent to which the observed patterns are generalizable across countries in the region. The specific objective are to:

(i) examine the relationship between urban poverty and HIV prevalence in SSA;
(ii) determine the extent to which the observed association between urban poverty and HIV prevalence varies across countries in the region; and
(iii) compare the risk factors of HIV seropositivity among the urban poor and non-poor

## Data and Methods

### The Data

This paper is based on secondary analysis of existing data from the international Demographic and Health Surveys (DHS) programme. The analysis uses data from the DHS and AIDS Indicator Surveys (AIS) collected between 2003 and 2008 from a total of 20 countries in SSA. A summary of the data analysed, classified by urban/rural residence and gender is given in Table 1.

**Table 1:** Summary of DHS in SSA analysed classified by urban/rural residence and gender

Country	Urban		Rural	
	Women	Men	Women	Men
Burkina Faso 2003	936	767	3253	2574
Cameroon 2004	2482	2462	2672	2579
Cote d’Ivoire 2005^a^	1972	1467	2563	2426
DR Congo 2007	2221	1908	2411	2396
Ethiopia 2005	1628	1158	4314	3949
Ghana 2003	2178	1515	3111	2750
Guinea 2005	1130	989	2712	1936
Kenya 2003	981	847	2290	2070
Lesotho 2004–2005	739	477	2281	1757
Liberia 2007	2878	2105	3604	3101
Malawi 2004	373	352	2491	2052
Mali 2006	1682	1377	3061	2509
Niger 2006	1474	1291	2967	1941
Rwanda 2005	1277	1071	4386	3657
Senegal 2005	2234	1847	2828	1913
Sierra Leone 2008	1427	1247	2039	1762
Swaziland 2006	1321	1155	3263	2447
Tanzania 2003–2004^a^	1548	1102	4421	3672
Zambia 2007	2536	2188	3177	2973
Zimbabwe 2005–2006	2448	1690	5046	3865
All (Sub-Saharan Africa)	33465	27015	62890	52329

^a^AIDS Indicator Survey (AIS)

The surveys presented in Table 1 comprised nationally representative samples of women and men of reproductive age (women aged 15–49 and males aged 15–54/59). Details of the sampling design and data collection procedures for each survey are available in the individual country DHS or AIS reports. The comparative nature of the DHS and AIS surveys makes it possible to pool data across countries to understand general patterns across the SSA region as well as cross-national variations.

The availability of HIV test data that can be linked to individual level survey data from recent DHS surveys, provides a unique opportunity for population-based studies of factors associated with the HIV/AIDS epidemic in different contexts. The DHS or AIS HIV testing protocol undergoes a rigorous ethical review process [27], providing for informed, anonymous, and voluntary testing of women and men of reproductive age.

### Methods of Analysis

The analysis starts with a bivariate examination of the association between poverty and HIV infection by urban/rural residence in each country included in the analysis. This is followed with a multivariate analysis of the urban poverty risk factor across countries in SSA, while simultaneously controlling for the effects of other important factors known to be associated with the risk of HIV infection. The analysis makes particular reference to urban poverty, through comparisons of the urban versus rural poverty as a risk factor for HIV prevalence, and an examination of urban SES inequalities in HIV infection.

The multivariate analysis involves application of multilevel logistic regression models applied to pooled DHS data from 20 countries in SSA. The modelling places particular emphasis on country and regional variations in factors associated with HIV prevalence in urban settings, and the extent of clustering of HIV positive individuals within countries and regions (i.e. provinces). The pooled data have a hierarchical structure with individuals nested within regions which are in turn nested within countries. In the multilevel analysis applied in this paper, countries constitute the highest (third) level (*n* = 20), while regions within countries constitute the second level. The general form of the three-level logistic regression model used may be expressed as:

1 $$ Logit  \pi_{ijk} = \, X_{ijk}^{\prime} b + Y_{ijk}^{\prime } u_{jk} +  Z_{ijk}^{\prime } v_{k} $$

where *π*_*ijk*_ is the probability of HIV positivity for an individual *i*, in the *j*th region in the *k*th country; $X_{ijk}^{\prime }$ is the vector of covariates which may be defined at the individual, region or country level; β is the associated vector of usual regression parameter estimates; $Y_{ijk}^{\prime }$ is a vector of covariates (usually a subset of $X_{ijk}^{\prime }$) which vary randomly at region level; $Z_{ijk}^{\prime }$ is a vector of covariates (usually a subset of $X_{ijk}^{\prime }$) which vary randomly at country level; and the quantities *v*_*k*_, and *u*_*jk*_ are the residuals at the country and region level, respectively. These are assumed to have normal distributions (with mean zero and variances *σ*_*v*_^2^ and *σ*_*u*_^2^) [28]

We have used the estimates of country and region level variances to derive intra-unit correlation coefficients to examine the extent to which the risk of HIV positivity is clustered within countries (or regions within countries) in urban settings of SSA, after taking into account the effect of significant covariates. Since individuals within the same region are also within the same country, the intra-region correlation includes country variances [29]. Thus, the intra-region (*ρ*_*u*_) and intra-country (*ρ*_*v*_) correlation coefficients are, respectively, given by:

2 $$ \mathop \rho \nolimits_{u} = \frac{{\mathop \sigma \nolimits_{u}^{2} + \mathop \sigma \nolimits_{v}^{2} }}{{\mathop \sigma \nolimits_{v}^{2} + \mathop \sigma \nolimits_{u}^{2} + \mathop \sigma \nolimits_{e}^{2} }} $$

and

3 $$ \mathop \rho \nolimits_{v} = \frac{{\mathop \sigma \nolimits_{v}^{2} }}{{\mathop \sigma \nolimits_{v}^{2} + \mathop \sigma \nolimits_{u}^{2} + \mathop \sigma \nolimits_{e}^{2} }} $$

where σ_v_^2^ is the total variance at country level; σ_u_^2^ is the total variance at region (i.e. province) level; and σ_e_^2^ is the total variance at individual level

We have assumed that the level-1 residuals, e_ijk_, for multilevel logistic regression model have a standard logistic distribution with mean zero and variance π^2^/3 (where π is the constant 3.1416) [30].

We note that the issue of a sufficient sample size is an important consideration in multilevel analysis. Although a number of studies have addressed the issue of what constitutes a sufficient sample size in multilevel models, a consensus has yet to develop [31–33]. Although the number of individuals per region or country in this paper is relatively large, the small number of Level-3 units (*n* = 20 countries) is likely to lead to reduced statistical power to detect significant country-level effects. It has been pointed out that power for individual-level estimates depends on the number of individuals, while power for higher level estimates depends on the number of groups [34, 35]. Thus, it is important to note that the relatively small number of countries in our analysis limits the inclusion of potentially important contextual country-level factors in the model. The analysis was undertaken using MLwiN multilevel software and estimations based on second order Predictive Quasi-Likelihood (PQL) procedure [36].

The key explanatory variable of interest is poverty (details of poverty measurement are given below), with particular reference to urban settings in SSA. In the multilevel models, the association between poverty and HIV infection is allowed to vary randomly at regional and country levels to establish the extent to which the observed overall patterns in the relationship between urban poverty and HIV infection varies between high and low HIV prevalence countries.

Besides poverty, a number of covariates, known to be associated with HIV prevalence, are included in the models as control variables. These include a range of background demographic, socio-economic and cultural characteristics, namely: gender, age, educational attainment, gender of household head and religion. In addition to the background factors, a set of sexual behavior risk factors are included in successive stages to establish possible pathways through which poverty is associated with HIV prevalence. These include current marital status, age at first marriage, age at first sex, premarital sex, non-use of condoms with non-spousal partner and multiple sexual partners.

### Poverty Measurement

It has been pointed out that as a measure of economic status, wealth (or its equivalent, net assets) has a number of advantages over income or expenditure: it represents a more permanent status; it is more easily measured (with only a single respondent needed in most cases); and it requires far fewer questions than either consumption expenditures or income [37, 38]. We have used the derived household wealth/asset index from the DHS data sets based on the work of Rutstein and Johnston [37]. This measure is based on Principal Components Analysis (PCA), a powerful tool in identifying the underlying patterns in the data and reducing the number of dimensions without much loss of information [39].

In this paper, the resulting PCA scores have been used to classify the rural and urban populations in each country into two equal halves: poor and non-poor, corresponding to the lower and upper 50 %, respectively. Thus, those below the median household wealth index score are classified as being poor, while those above the median are classified as non-poor. Rutstein and Johnston [37, p. 6] noted that ‘a poverty line based on a national percentile distribution of households by economic status, such as wealth quintiles, is useful in assessing the reach of public health programs for both the poorer and richer sections of society’. Our poverty threshold to define those in poverty has been set at a much higher level than the standards commonly used for relative poverty measurement in developed country settings (where the poor are often classified as those in households below 60 % of median income) in recognition of the fact that more than half of the urban or rural populations in most of SSA live in poverty. For instance, the UNDP estimates based on the most recent data available during 2000–2007 suggest that about half of the countries included in this study have more than half their population classified as living below the national poverty lines [40, p. 33].

Our measure of poverty is therefore a relative, rather than an absolute poverty measure. The index is relative within urban and rural environments in each country to account for potential urban/rural differences across countries in the meaning of household assets and amenities used to derive the index.^1^ The disparity in what constitutes wealth across different cultural and social contexts is exemplified by the considerable national variations in the distribution of the population in different settings who possess various assets by wealth index [37, pp 19–23].

## Results

A bivariate analysis of the association between poverty and HIV prevalence by urban/rural residence in individual countries suggests that while HIV prevalence in urban areas is generally higher among the poor than non-poor, this pattern is reversed in rural areas (Table 2). In all the countries where there is a significant association between poverty and HIV infection, the results suggest that in urban areas, the poor are more likely to be infected with HIV than the non-poor, while in rural areas, it is the non-poor who are more likely to be infected with HIV (except in Swaziland).

**Table 2:** Association between poverty and HIV infection by urban/rural residence

Country	Percent HIV positive					
	Urban		Sig.	Rural		Sig.
	Non-poor	Poor		Non-poor	Poor	
Burkina Faso 2003	4.1	3.7	ns	1.0	1.5	ns
Cameroon 2004	6.6	6.2	ns	5.4	2.6	***
Cote d’Ivoire 2005^1^	5.9	4.8	ns	5.0	2.9	***
DR Congo 2007	1.9	1.8	ns	0.9	0.7	ns
Ethiopia 2005	4.5	6.1	ns	0.7	0.6	ns
Ghana 2003	2.0	2.6	ns	2.6	1.4	**
Guinea 2005	2.0	3.5	*	0.8	1.1	ns
Kenya 2003	9.4	10.6	ns	5.9	5.3	ns
Lesotho 2004–2005	26.0	32.9	*	22.3	20.8	ns
Liberia 2007	2.8	2.1	ns	1.1	0.8	ns
Malawi 2004	15.9	18.4	ns	13.1	8.3	***
Mali 2006	2.0	1.4	ns	1.0	1.2	ns
Niger 2006	1.4	1.6	ns	0.5	0.5	ns
Rwanda 2005	8.0	6.1	ns	2.3	1.9	ns
Senegal 2005	0.6	0.7	ns	0.7	0.7	ns
Sierra Leone 2008	2.4	2.4	ns	0.9	1.0	ns
Swaziland 2006	25.4	36.5	***	22.1	25.7	**
Tanzania 2003–2004^a^	9.9	12.0	ns	6.7	3.8	***
Zambia 2007	18.1	21.7	**	11.8	8.8	***
Zimbabwe 2005–2006	14.3	23.2	***	18.1	17.1	ns
All (Sub-Saharan Africa)	7.3	9.2	***	6.2	5.3	***

* Statistical significance (Chi-Square test) at 5 % level—*p* < 0.05; ** at 1 %—*p* < 0.01; and *** at 0.1 %—*p* < 0.001

The multilevel logistic regression analysis first examined the urban poverty risk factor in HIV prevalence across regions and countries in SSA, using pooled DHS data from 20 countries in SSA. A comparison of the risk of poverty in HIV infection in urban and rural areas of SSA is presented in Table 3, including estimates for the other significant covariates in the model. The results for Model 1 include only the background characteristics while sexual behaviour factors are introduced in Model 2 to establish the extent to which the observed risk of poverty may be explained by differences in sexual behaviour factors between the poor and non-poor.

**Table 3:** Multilevel logistic regression parameter estimates of HIV infection by urban/rural residence

Parameter (reference category in brackets)	Urban		Rural	
	Model 1	Model 2	Model 1	Model 2
Fixed effects				
Constant	−2.85(0.268)	−3.25(0.271)	−3.17(0.292)	−3.81(0.288)
Poverty status (non-poor)				
Poor	0.17(0.037)*	0.13(0.037)*	−0.15(0.030)*	−0.18(0.064)*
Gender (female)				
Male	−0.43(0.037)*	−0.51(0.043)*	−0.36(0.031)*	−0.42(0.037)*
Age group (45+)				
15−19	−1.62(0.086)*	−0.83(0.101)*	−1.74(0.070)*	−0.94(0.083)*
20−24	−0.49(0.072)*	−0.12(0.079)	−0.39(0.051)*	−0.09(0.062)
25−29	0.24(0.069)*	0.43(0.072)*	0.22(0.055)*	0.41(0.057)*
30−34	0.56(0.069)*	0.66(0.072)*	0.49(0.055)*	0.63(0.056)*
35−39	0.62(0.072)*	0.69(0.074)*	0.52(0.057)*	0.60(0.058)*
40−44	0.43(0.077)*	0.47(0.079)*	0.36(0.060)*	0.41(0.062)*
Education level (none)				
Primary	0.24(0.065)*	0.21(0.065)*	0.22(0.045)*	0.19(0.046)*
Secondary	0.02(0.066)	0.04(0.067)	0.22(0.051)*	0.21(0.053)*
Sex of household head (male)				
Female	0.39(0.039)*	0.16(0.044)*	0.34(0.033)*	0.07(0.036)*
Religion (Catholic/Orthodox)				
Protestant/otherChristian	−0.07(0.048)	−0.06(0.049)	−0.08(0.044)	−0.07(0.045)
Muslim	−0.28(0.076)*	−0.24(0.077)*	−0.23(0.076)*	−0.21(0.076)*
Other/none	−0.01(0.082)	−0.05(0.084)	−0.01(0.060)	0.02(0.061)
Marital status (married−mono)				
Never married		0.00(0.076)		0.16(0.068)*
Married-polygamous		0.06(0.073)		0.01(0.051)
Widowed		1.21(0.074)*		1.31(0.060)*
Divorced/separated		0.57(0.064)*		0.73(0.056)*
Age at first marriage (20+)				
<16 years		0.01(0.087)		−0.00(0.071)
16–17		−0.18(0.072)*		−0.15(0.057)*
18–19		−0.14(0.062)*		−0.09(0.049)
Age at first sex (20+)				
Never had sex		−0.83(0.104)*		−0.92(0.096)*
<16 years		0.13(0.064)*		0.05(0.057)
16–17		0.23(0.057)*		0.14(0.051)*
18–19		0.10(0.054)		0.14(0.047)*
Premarital sex		0.31(0.054)*		0.34(0.043)*
Non-condom use^$^		0.17(0.053)*		0.08(0.048)
Multiple sex partners		0.35(0.057)*		0.24(0.052)*
Random effects				
Region—constant	0.12(0.023)*	0.11(0.022)*	0.14(0.021)*	0.20(0.033)*
Country—constant	1.25(0.405)*	1.23(0.398)*	1.38(0.460)*	1.48(0.484)*

Standard errors given in *brackets*

Model 1—controlling for background socio-economic and demographic factors

Model 2—controlling for background factors plus sexual behaviour factors

* Statistical significance at 5 % level—*p* < 0.05 (*Z* test based on ratio of estimate to its standard error)

^$^No condom use at last sex, with non-spousal partner

The results from the multilevel logistic regression analysis confirm patterns from the bivariate analysis. Across countries in SSA, the urban poor have on average a 19 % (i.e. exp(0.17)) *higher* odds of being HIV positive than their non-poor counterparts of similar background characteristics with respect to gender, age group, educational attainment, sex of household head and religious affiliation (Model 1). On the other hand, the rural poor have on average a 14 % (i.e. exp(−0.15)) *lower* odds of being HIV positive than their non-poor counterparts of similar background characteristics.

An examination of potential pathways through which HIV prevalence may be linked to urban poverty, suggests that the urban poor disadvantage persists even after sexual behaviour factors relating to current marital status, age at first marriage, age at first sex, premarital sex, non-condom use with non-spousal partner, and multiple sex partnerships are controlled for. On average the urban poor have 14 % *higher* odds of being HIV positive than their non-poor counterparts with similar sexual behaviour. For rural residents, the poor have 16 % *lower* odds compared to their non-poor counterparts of similar sexual behaviour. Thus, controlling for sexual behaviour factors tends to lower the relative risk of HIV prevalence among the poor, both in urban and rural areas.

The random effects estimates show significant variations in HIV prevalence across countries and to a lesser extent across regions within countries in urban and rural settings of SSA. The estimates of intra-unit correlations suggest that 27 % of the total unexplained variation in HIV prevalence in urban settings is attributable to unobserved country level factors, which is about the same, albeit slightly lower than in rural areas (30 %). Although the risk of poverty was allowed to vary randomly at regional and country levels, there was no evidence of significant variations in the association between poverty and HIV prevalence across countries or regions within countries. However, we need to exercise caution in interpreting the lack of significance across countries from the multilevel models given the small number of countries (*n* = 20) and consequent reduced statistical power to detect significant factors at this level.

Next, we focus on the risk factors of HIV positivity among the urban poor, the sub-group associated with the highest HIV prevalence. A comparison of the risk factors of HIV prevalence among the urban poor and urban non-poor suggests that overall, the risk factors among the urban poor and urban non-poor are more or less similar, but with some exceptions (Table 4). One of the notable differences relates to the gender disparity in HIV positivity which is greater among the urban poor than urban non-poor. Across countries in SSA, urban poor women have about double the odds of being HIV positive than urban poor men, while among the urban non-poor, the average odds for women is about 1.5 times higher than that of male counterparts.

**Table 4:** Multilevel logistic regression parameter estimates of HIV infection among the urban poor and urban non-poor in sub-Saharan Africa (Standard Errors in brackets)

Parameter	Urban poor	Urban non-poor
Fixed effects		
Constant	−3.17(0.289)	−2.95(0.296)
Gender (female)		
Male	−0.61(0.062)*	−0.41(0.060)*
Age group (45+)		
15–19	−0.92(0.140)*	−0.74(0.146)*
20–24	−0.15(0.108)	−0.14(0.117)
25–29	0.35(0.100)*	0.50(0.105)*
30–34	0.65(0.099)*	0.66(0.104)*
35–39	0.66(0.103)*	0.72(0.106)*
40–44	0.44(0.110)*	0.50(0.114)*
Education level (none)		
Primary	0.29(0.083)*	0.02(0.109)
Secondary +	0.22(0.088)*	−0.29(0.107)*
Sex of household head (male)		
Female	0.20(0.061)*	0.11(0.064)
Religion (catholic/orthodox)		
Protestant/other Christian	− 0.12(0.070)	0.01(0.068)
Muslim	−0.34(0.105)*	−0.17(0.112)
Other/none	−0.06(0.112)	−0.09(0.129)
Marital status (married-monogamous)		
Never married	0.14(0.108)	−0.17(0.108)
Married -polygamous	0.04(0.097)	0.11(0.113)
Widowed	1.22(0.100)*	1.19(0.112)*
Divorced/separated	0.61(0.087)*	0.53(0.094)*
Age at first marriage (20+)		
<16 years	0.08(0.117)	−0.08(0.135)
16–17	−0.13(0.096)	−0.23(0.112)*
18–19	−0.11(0.085)	−0.18(0.093)
Age at first sex (20+)		
Never had sex	−1.05(0.159)*	−0.67(0.138)*
<16 years	0.02(0.090)	0.21(0.093)*
16–17	0.12(0.081)	0.31(0.082)*
18–19	0.01(0.078)	0.17(0.076)*
Premarital sex	0.47(0.074)*	0.14(0.079)
Non-condom use^$^	0.21(0.072)*	0.08(0.079)
Multiple sex partners	0.30(0.091)*	0.38(0.082)*
Random effects		
Region—constant	0.14(0.032)*	0.06(0.022)*
Country—constant	1.27(0.414)*	1.24(0.406)*

* Statistical significance at 5 % level—*p* < 0.05 (*Z* test based on ratio of estimate to its standard error)

^$^No condom use at last sex, with non-spousal partner

Another notable difference between the urban poor and urban non-poor relates to educational attainment. It is interesting to note that while higher educational attainment is associated with reduced odds of HIV positivity among the urban non-poor, it is associated with increased odds among the urban poor. Among the urban poor, those with at least secondary level education have on average a 25 % higher odds of being HIV positive than their counterparts of similar characteristics with no formal education, while among the urban non-poor, the odds are on average 25 % lower. The association between HIV prevalence and other background characteristics such as gender of household head and religious affiliation also tend to be stronger among the urban poor than urban non-poor.

With respect to sexual behaviour factors, premarital sex and no condom use during last sex with non-spousal partner are both associated with significantly higher odds of HIV positivity among the urban poor, but not among the urban non-poor. In particular, premarital sex is associated with a 60 % increase in the odds of HIV positivity among the urban poor, but there is no significant difference among the urban non-poor by whether or not they had premarital sex. While sexual abstinence is more protective among the urban poor than urban non-poor, early sexual debut is not a significant risk factor among the urban poor, albeit significant among the urban non-poor. Also, it is interesting to note that early marriage is protective among the urban non-poor but not significant among the urban poor, when age at first sex is controlled for.

Overall, there are significant variations in HIV prevalence across countries (and to a lesser extent across regions within countries) among both the urban poor and urban non-poor.

## Discussion and Conclusions

One important data limitations should be borne in mind when interpreting the findings of this study. This relates to our inability to infer precise causal relationships, given the cross-sectional nature of data analysed.^2^ Therefore, the relationships between HIV prevalence and poverty observed are mere associations rather than causal relationships.

This study confirms that the public health consequences of urban poverty under conditions of rapid urban growth that has been well documented in previous studies with respect to child or maternal health do apply to the risk of HIV positivity. The urban poor in SSA do experience comparative disadvantage with respect to HIV prevalence. While HIV prevalence is significantly *lower* among the poor than non-poor in rural areas, it is significantly *higher* among the urban poor than urban non-poor. Thus, the positive association between household wealth and HIV prevalence observed in previous studies [6–8] largely reflects the situation in the rural areas where the majority of the populations in SSA reside.

The observed higher risk of HIV among the urban poor is consistent with Holmqvist’s interpretation grounded in a theory of economics of sexual behavior, arguing that the adverse future chances of people living in poverty is likely to increase their readiness to take risks today [11]. The fact that the observed disadvantage among the poor is observed only among urban and not rural residents may be partly attributable to weaker social cohesion in urban settings. Holmqvist emphasized the role of social cohesion in establishing norms, communicating with trust and mobilizing collective resources in the pursuit of joint goals or to control risk. The fact that the urban poor disadvantage persists even after sexual behavior factors relating to current marital status, age at first marriage, age at first sex, premarital sex, non-use of condoms/type of partner, and multiple sex partners are controlled for suggests that the observed differences cannot be fully explained by these sexual behavior factors. Perhaps factors characterizing the urban poor in most of SSA such as unemployment, discrimination, violence, and crime that have been previously shown to relate closely to HIV/AIDS risks [24] play an important role.

Although low education has been noted as one of the factors related to HIV/AIDS risk [25], it is unlikely to contribute to the higher risk of HIV prevalence observed among the urban poor in SSA. Indeed, our findings suggest that while higher educational attainment is associated with a reduced risk of HIV positivity among the urban non-poor, the association is reversed among the urban poor.

Our analysis placed particular interest on gender disparities in the poverty and HIV relationship, especially since it has been noted that poverty places women at a special disadvantage [10]. However, an interaction between gender and poverty included in our analysis was not significant, suggesting that the relationship between HIV and poverty does not vary significantly between men and women. An important related finding, however, refers to increased vulnerability among urban poor women. While the odds of being HIV positive for urban non-poor or rural women is 1.5 times higher than their male counterparts of similar characteristics, the odds are about double for urban poor women than urban poor men of similar characteristics. The eminent vulnerability among urban poor women is further demonstrated by the higher risk of HIV positivity among the urban poor in female headed households, a disadvantage that persists even after the expected higher risk among those who are widowed, divorced or separated (who comprise a disproportionately high proportion of women in female headed households) are controlled for. It is possible that factors such as unemployment, discrimination, violence and crime that typify the urban poor in SSA may indeed increase women’s vulnerabuility to HIV infection. Rodrigo and Rajapakse [10] explain how poverty may interact with other non-biological factors such as violence or gender inequality to increase the HIV risk for women. This explanation is consistent with findings from studies among the urban poor in South Africa that highlight increased women’s vulnerability to the risk of HIV due to transactional sex that is associated with gender-based violence and socio-economic disadvantage [14, 15].

The findings relating to gender disparities in the poverty and HIV relationship have important policy/programme implications. The increased vulnerability among urban poor women compared to their male counterparts of similar characteristics underscores the important role of interventions aimed at addressing factors such as gender violence, inequality and discrimination in tackling increased women’s vulnerability to HIV infection among the urban poor in SSA.

The observed patterns with respect to sexual behavior risk factors among the urban poor and urban non-poor also have important policy/programme implications. In particular, the fact that sexual abstinence is particularly protective and that premarital sex is a strong risk factor for HIV positivity among the urban poor underscores the importance of interventions to reduce the incidence of premarital sex among this population sub-group. While earlier sexual debut is a risk factor among the urban non-poor, for the urban poor premarital sex is a more important risk factor. Efforts to reduce the risk of HIV infection among the urban poor should take note of the fact that premarital sexual activity among the urban poor involves particularly high risk, regardless of the timing of sexual debut.

Perhaps of greater policy/programmatic significance is the finding relating to non-use of condoms in casual partnerships, which is a significant risk factor among the urban poor but not among the urban non-poor or rural residents. This underscores the importance of interventions aimed at increasing access to, and utilization of, condoms among the urban poor.

Overall, HIV prevalence among the urban poor in SSA varies significantly across countries and to a lesser extent across regions (i.e. provinces) within countries. However, there is no evidence that the observed urban inequalities in SSA vary significantly across countries, suggesting that the observed patterns may be generalized across countries in the region.

## Footnotes

1 The indicator variables used to derive the wealth/poverty index comprised a wide range of household assets and utility services, including country-specific items. The determination of specific indicator variables depended on knowledge of conditions in each country, which sometimes involved removal of particular items from the set of indicators in order for the resulting index to be meaningful [37].

2 We recognize that poverty and HIV infection are intrinsically linked and while our focus in this paper is on the role of poverty or wealth in bringing about risky social interaction that raises the chance of contracting HIV, we recognize that HIV infection may also increase the risk of poverty among affected individuals, households and communities. Given the cross-sectional nature of data analysed, we are unable to establish the time sequencing of events of interest: that is, whether the current poverty status preceded HIV infection or a consequence of it.
